# Case Report: Delayed diagnosis of parathyroid carcinoma and two pulmonary recurrences and metastases

**DOI:** 10.3389/fonc.2025.1581911

**Published:** 2025-09-24

**Authors:** Wenzhi Tian, Chenchen Hu, Tianmin Cao, Dong Chen, Xi Su, Peng Li

**Affiliations:** ^1^ Department of Thyroid and Breast Surgery, Peking University Shenzhen Hospital, Peking University-The Hong Kong University of Science and Technology Medical Centre, Shenzhen, Guangdong, China; ^2^ Shenzhen University Health Science Center, Shenzhen, Guangdong, China

**Keywords:** parathyroid carcinoma, delayed diagnosis, standardized surgery, hypercalcemia, high PTH, lung metastasis

## Abstract

Parathyroid carcinoma (PC) is a rare and aggressive malignancy, characterized by severe hypercalcemia and elevated parathyroid hormone (PTH) levels, making it particularly challenging to diagnose. In this case report, the patient’s PC was not diagnosed during the initial surgery. The diagnosis was delayed until two years later, when metastatic lesions appeared in the lungs, leading to repeat surgery and a retrospective review of the patient’s medical history. This case emphasizes the diagnostic difficulty of PC, particularly during the initial evaluation. Close postoperative follow-up is crucial for suspected cases. Upon biochemical evidence of recurrence, comprehensive systemic screening should be conducted, not only focusing on the neck but also on common metastatic sites such as the lungs, bones, and liver.

## 1 Introduction

Parathyroid carcinoma (PC) is a rare endocrine malignancy, representing less than 1% of primary hyperparathyroidism (PHPT) cases (1). The clinical presentation of PC often resembles benign hyperparathyroid conditions, such as parathyroid adenomas, which makes early diagnosis particularly challenging. Hallmark features of PC include persistent hypercalcemia, markedly elevated parathyroid hormone (PTH) levels, and, in some instances, a palpable neck mass (2). The potential for local invasion and distant metastasis distinguishes PC from its benign counterparts. Early detection is critical for effective treatment (3).

The diagnosis of PC is often delayed due to symptom overlap with benign conditions. Key signs like high PTH levels, neck masses, or severe hypercalcemia can indicate PC. Skilled parathyroid surgeons can identify these signs, make a presumptive diagnosis, and plan surgery accordingly. Early detection and tailored surgical planning are crucial, as complete resection provides the best chance for disease control and long-term management. Therefore, maintaining a high index of suspicion and relying on both clinical and biochemical markers are paramount in guiding treatment decisions for suspected PC cases.

Additionally, pulmonary metastasis, although uncommon, is a well-recognized complication in advanced PC. Distant metastases occur in 25-30% of cases and often involve the lungs (40%), bones, and liver (4, 5). Surgery remains the primary treatment modality for both primary tumors and metastatic lesions, offering the best chance for disease control and symptom relief (6, 7). However, recurrence is common, and long-term prognosis remains guarded. This case report describes a patient with PC who developed two episodes of pulmonary metastasis, in 2018 and 2023, both of which were successfully managed with surgical resection. The case underscores the importance of continuous surveillance and emphasizes the role of surgery in managing metastatic PC, particularly with pulmonary involvement.

## 2 Case presentation

A 30-year-old female patient came to our hospital in May 2016 because she accidentally discovered a left-sided neck mass one year ago, accompanied by symmetrical numbness of the whole body, dyspnea and swallowing, and pain behind the ear. Routine blood tests revealed hypercalcemia with a serum calcium level of 12.2 mg/dL (3.05 mmol/L) (normal reference range: 8.5-10.2 mg/dL; 2.12-2.55 mmol/L) and an elevated intact PTH level of 206 pg/mL (19.5 pmol/L) (normal reference range: 15-65 pg/mL; 1.6-6.9 pmol/L), leading to the definitive diagnosis of primary hyperparathyroidism.

**Figure 1:**
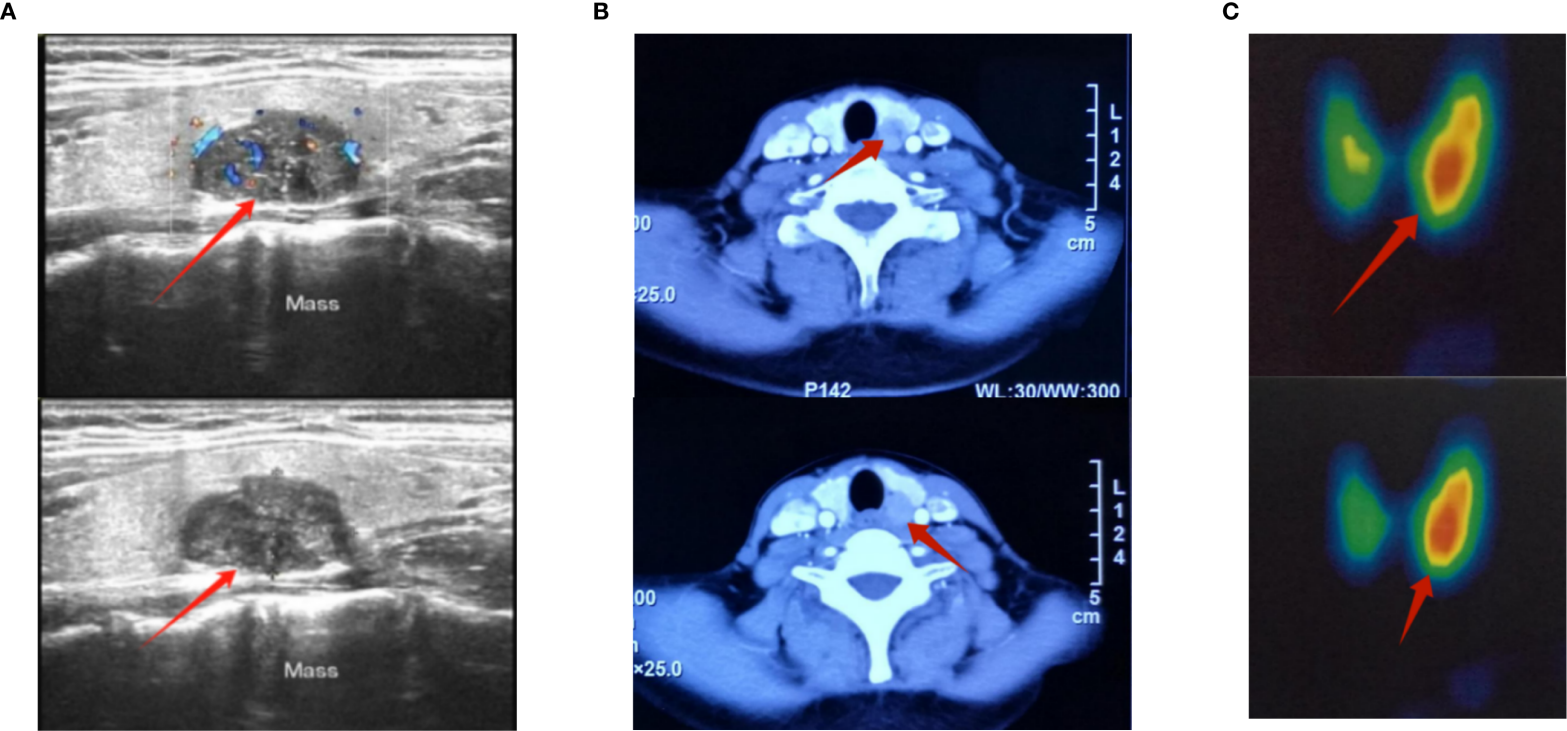
Preoperative color Doppler ultrasound, CT and 2.99mTc-MIBI images of the neck for parathyroid tumor. **(A)** Preoperative color Doppler ultrasound image of the parathyroid gland; the red arrow marks the location of the tumor. The diagnosis showed a solid mass in the left lobe of the thyroid gland, measuring 27×21×17 mm, of unknown nature. **(B)** Preoperative CT image of parathyroid gland, the red arrow marks the location of the tumor. The result showed a nodule in the inner and posterior part of the left thyroid lobe, measuring 14×13×17mm, which was considered to be an adenoma. **(C)** Conventional thyroid imaging before parathyroid surgery 2.99mTc-MIBI thyroid-parathyroid imaging showed: Parathyroid imaging (digital subtraction imaging) showed a radioactive distribution concentration focus in the area equivalent to the left lower parathyroid gland, with a size of about 1.5×2.0cm. There was no obvious abnormality in the position of the thyroid gland, which still maintained a “butterfly” shape. The bilateral lobes were not large in shape, and the radioactive distribution was uneven. The asymmetric uptake rate (%): right lobe = 0.4, left lobe = 0.6, total: 1.0. Area (Cm²): right lobe = 7.9, left lobe = 9.4, total: 17.3. Digital subtraction imaging showed the imaging findings of left lower parathyroid adenoma and hyperfunction; the red arrow marks the location of the tumor.

**Figure 2:**
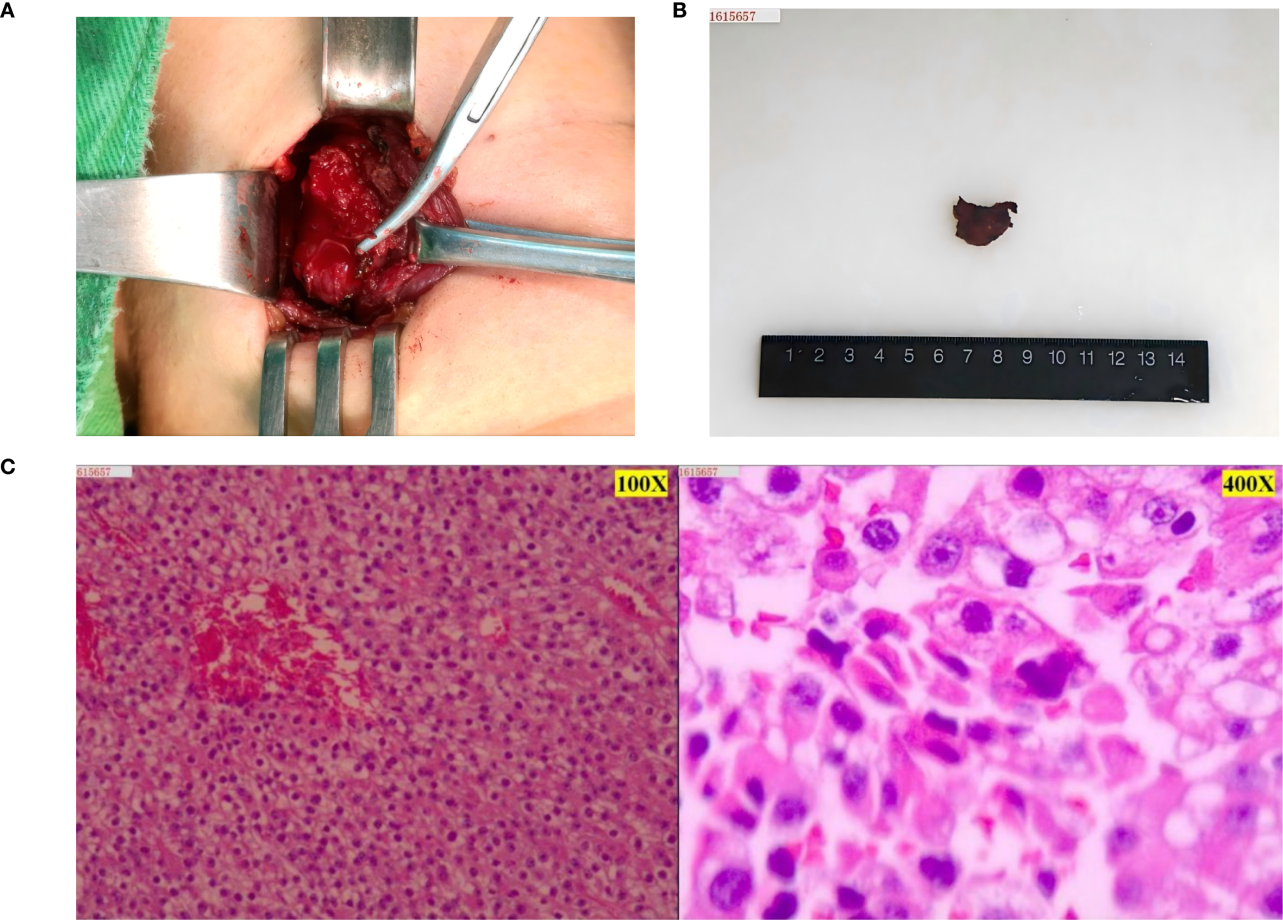
Intraoperative and postoperative tumor and postoperative pathological images of parathyroid tumor. **(A)** Parathyroid tumor seen during surgery. **(B)** Gross image of a parathyroid tumor removed surgically. **(C)** The first postoperative pathology showed an atypical parathyroid adenoma of the “left inferior parathyroid gland”, with focal extracapsular infiltration, and the possibility of malignancy could not be ruled out. Combined with the comprehensive clinical judgment, the current diagnosis was left inferior parathyroid adenocarcinoma (left side microscope was 100X, right side microscope was 400X). Immunohistochemistry results (positive and negative controls were set up): tumor cells: CgA (focal +); Sym (weak +); TTF1 (-); Ki-67 (about 15-20% +); CD34 (no vascular infiltration); Parafibromin (-).

Neck ultrasonography revealed a 2.8-cm hypoechoic mass, an intrathyroidal lesion located in the middle to lower portion of the left thyroid lobe. Additionally, both 2.99mTc-MIBI thyroid-parathyroid scintigraphy and neck CT confirmed the localization of the hyperparathyroid lesion (**Figure 1**). In June 2016, the patient underwent en bloc parathyroidectomy with left lobectomy and ipsilateral central neck dissection. During the operation, the glandular tissue was removed, and the intraoperative rapid freezing pathology report showed: left parathyroid tumor, cells with certain atypia, accompanied by necrosis, waiting for routine pathology and immunohistochemistry to exclude parathyroid cancer. Combined with the intraoperative findings of tumor adhesion to the thyroid gland, the possibility of parathyroid cancer was considered. Therefore, we performed left parathyroid tumor resection + left thyroidectomy + left central lymph node dissection + left recurrent laryngeal nerve exploration. After the operation, routine postoperative pathology suggested that the possibility of malignancy could not be ruled out. Combined with the clinical diagnosis, it was parathyroid adenoma. At the same time, the patient’s calcium and parathyroid hormone levels returned to normal, and she had no symptoms for the next two years (**Figure 2**).

**Figure 3:**
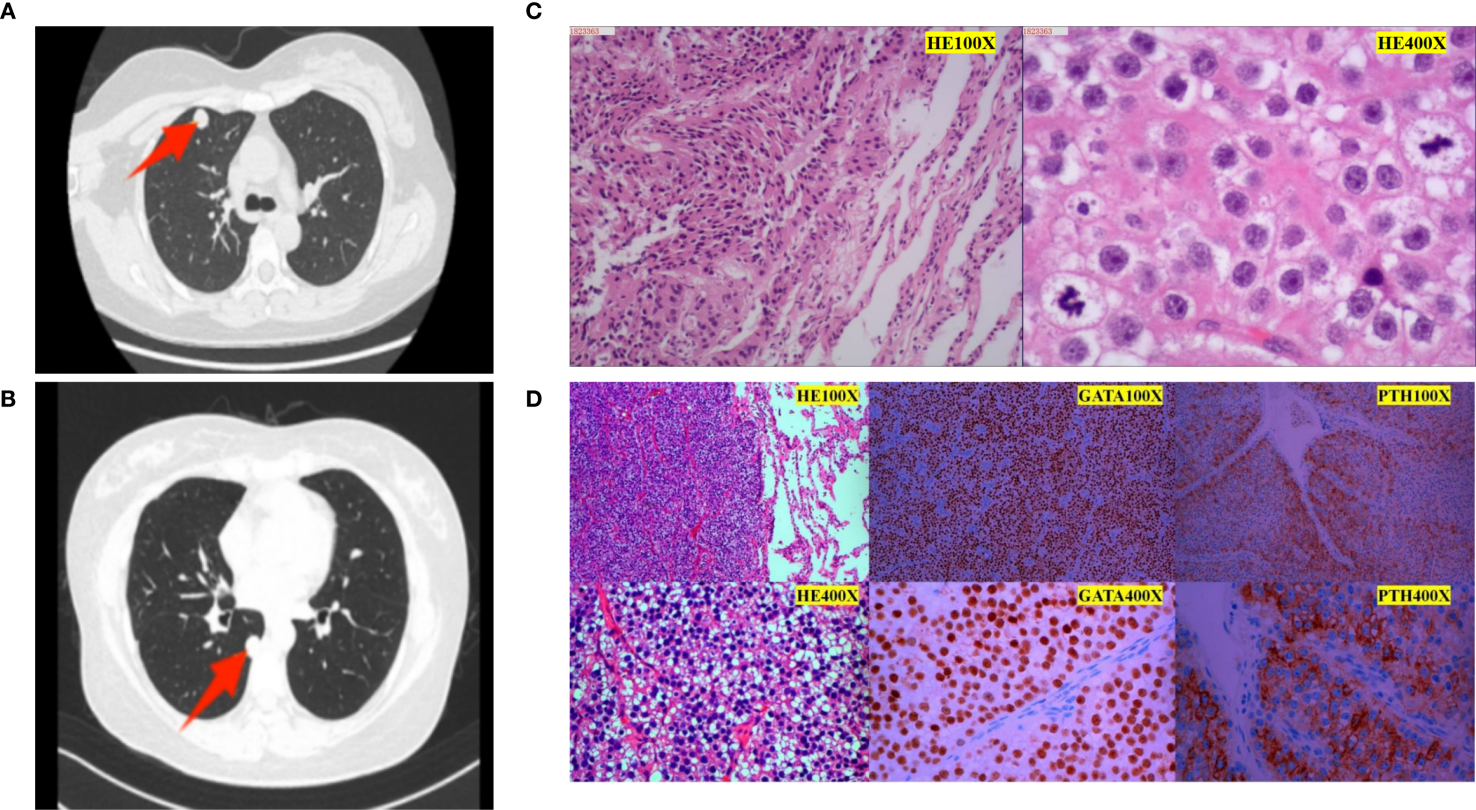
Imaging and pathological images of two pulmonary metastases. **(A)** Chest CT image before the first lung metastasis surgery. A nodule was seen in the anterior segment of the right upper lobe, measuring approximately 13 mm in diameter with smooth edges and obvious enhancement on enhanced scanning. The yellow arrow indicates the location of the tumor. **(B)** Chest CT image before the second lung metastasis surgery. A solid nodule was found under the pleura in the dorsal segment of the right lower lobe, approximately 15mmx12mm in size, the yellow arrow indicates the location of the tumor. **(C)** Pathology after the first lung metastasis surgery: The “right upper lung nodule” was suspected to be a metastatic parathyroid tumor(left side microscope was 100X, right side microscope was 400X). **(D)** Postoperative pathology of the second lung metastasis: The morphology of the “right lower lung dorsal segment and nodules” is considered to be a neuroendocrine tumor. Combined with immunohistochemistry and medical history, it is consistent with metastatic parathyroid tumor. Immunohistochemistry results (positive and negative controls were set up): tumor cell SSTR2 (scattered a little +), CgA (scattered a little +), SYN (scattered a little +), CD56 (-), CK-pan (+), Ki-67 (about 5% +), TTF1 (-), GATA-3 (+), Calcitonin (-), PTH (+).

In June 2018, during a routine follow-up, the patient was again found to have hypercalcemia serum calcium 13.4 mg/dL (3.36 mmol/L) (normal reference range: 8.5-10.2 mg/dL; 2.12-2.55 mmol/L) and elevated PTH levels 152 pg/mL (14.3 pmol/L) (normal reference range: 15-65 pg/mL; 1.6-6.9 pmol/L). At the same time, color Doppler ultrasonography of the thyroid gland and parathyroid gland in the neck showed no abnormalities. A chest CT scan detected a solitary 1.6 cm nodule in the right lower lobe of the lung, suggestive of suspicious metastatic lesions. The patient underwent video-assisted thoracoscopic surgery (VATS) for the resection of the lung nodule, which was histologically confirmed as a metastatic lesion from the parathyroid carcinoma. The patient remained stable until September 2023, when surveillance imaging identified one new nodule in the right lung (measuring 1.5 cm)(**Figure 3**). Laboratory tests showed recurrence of hypercalcemia 13.14 mg/dL (3.29 mmol/L) (normal reference range: 8.5-10.2 mg/dL; 2.12-2.55 mmol/L) and elevated PTH levels 181 pg/mL (17.2 pmol/L) (normal reference range: 15-65 pg/mL; 1.6-6.9 pmol/L). In October 2023, she underwent another VATS resection of the nodule, which were confirmed to be metastatic parathyroid carcinoma (**Figure 3**). The patient has since stabilized, with normal serum calcium and PTH levels as of her latest follow-up in January 2024. She continues regular monitoring, and no signs of further recurrence have been observed.

Because the patient had two lung metastases after the first parathyroidectomy, we reviewed the parathyroid pathology specimen of the patient’s 2016 lesion. Previous pathological slides were reviewed and immunohistochemical testing for parafibromin was performed. We selected normal parathyroid tissue and breast cancer tissue as positive controls and found that the nuclear expression of parafibromin was negative in this parathyroid lesion tissue (**Supplementary Figure 1**). We also performed mitotic count on HE-stained slides, and the mitotic count was 13/10 mm². On the basis of these findings, we can confirm that the patient should receive a diagnosis of parathyroid malignancy.

## 3 Diagnostic assessment

The patient was initially diagnosed with primary hyperparathyroidism based on clinical presentation: hypercalcemia, a palpable neck mass, and elevated parathyroid hormone levels, all suggestive of primary hyperparathyroidism. Imaging studies, including neck ultrasound and CT tomography, identified a left inferior parathyroid gland as the likely etiology of his condition. Malignancy was considered possible based on the intraoperative rapid pathology results and intraoperative PTH changes, as well as postoperative pathology with histopathological findings suggesting capsular and vascular invasion and increased mitotic figures, but was not confirmed.

After, regular biochemical monitoring and imaging studies are essential to detect disease recurrence and metastatic spread. In 2018, elevated calcium and parathyroid hormone levels raised suspicion of recurrence, and chest CT revealed pulmonary nodules, suggestive of metastatic spread. The decision to proceed with surgical resection of the pulmonary nodule was based on the possibility of curative intervention for isolated metastases. After the operation, based on the pathological results of the lung metastatic nodules and a review of the patient’s medical history, the patient was diagnosed with parathyroid carcinoma.

In 2023, imaging studies revealed new pulmonary nodules, and biochemical markers suggested disease recurrence, and a similar diagnostic approach was subsequently adopted. This further emphasizes the need for close surveillance of patients with parathyroid cancer, as metastatic disease may develop even years after initial treatment. Subsequent VATS surgery and pathological confirmation of metastatic parathyroid cancer are essential to guide further treatment and ensure continued monitoring of the patient’s condition.

Throughout her follow-up, regular biochemical evaluations, including serum calcium and parathyroid hormone levels, are essential to assess disease activity, guide imaging decisions, and therapeutic interventions. Integration of clinical, biochemical, and radiological data is essential to diagnose recurrent disease and determine appropriate treatment strategies.

## 4 Treatment

Surgical intervention was the primary treatment for managing both the primary parathyroid carcinoma and its metastases. In 2016, the patient underwent left parathyroidectomy, total thyroidectomy, and lymph node dissection. Complete removal of the tumor was critical due to its malignant nature and local invasion. In 2018, following the detection of pulmonary metastasis, a thoracoscopic wedge resection was performed, which effectively controlled the recurrence, as parathyroid carcinoma metastases are largely resistant to chemotherapy or radiation.

In 2023, a new lesion in the right lung required thoracoscopic segmental resection, confirming the metastatic nature of the disease. Surgical excision significantly reduced tumor burden and helped manage the patient’s hypercalcemia. Postoperatively, the patient was treated with calcium and vitamin D supplements, with close monitoring of serum calcium and PTH levels to detect recurrence and ensure disease control.

## 5 Outcome and follow-up

**Figure 4:**
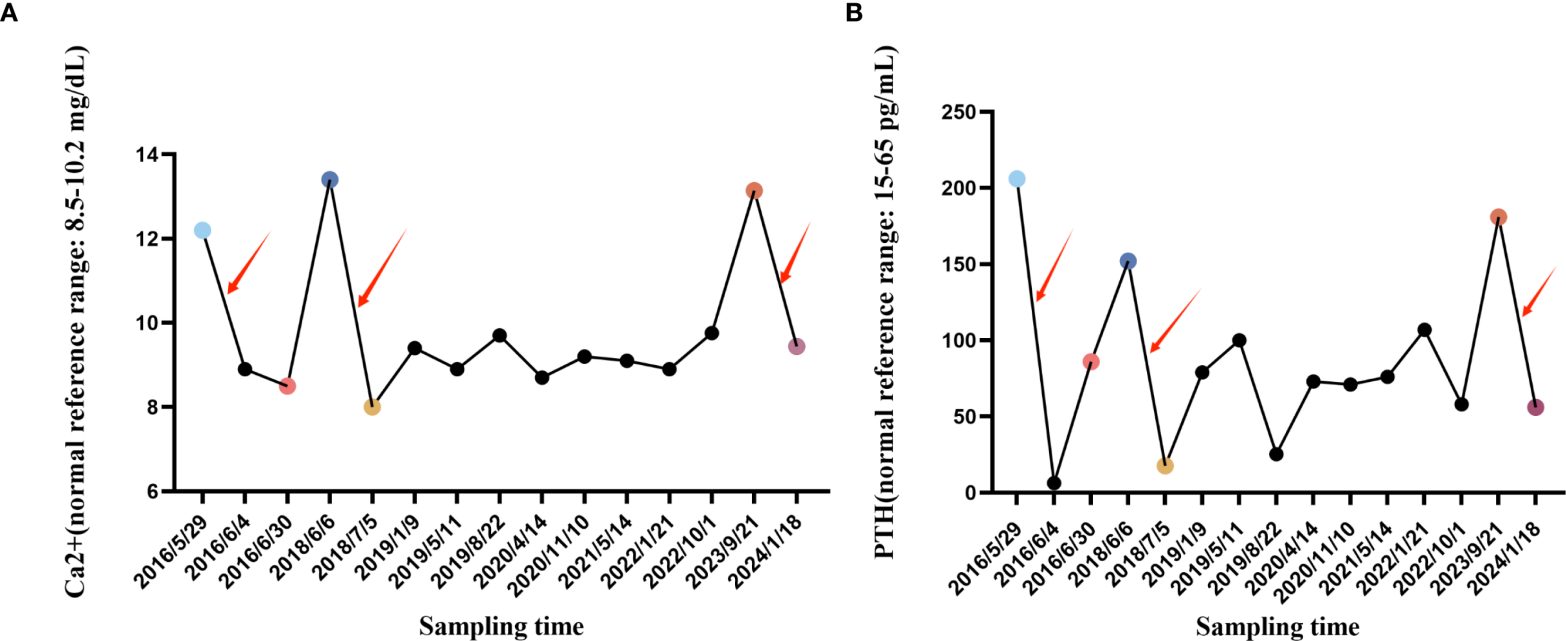
Line graph of changes in blood calcium and blood PTH. **(A)** The blood Ca2+ change curve during hospitalization and follow-up in our hospital from May 29, 2016 to January 18, 2024, where the specially marked points are the blood Ca2+ concentration before the first parathyroid cancer operation, the blood Ca2+ concentration after the first parathyroid cancer operation, the Ca2+ ion concentration before the first tumor lung metastasis, the blood Ca2+ concentration after the first tumor lung metastasis operation, the Ca2+ ion concentration before the second tumor lung metastasis, and the Ca2+ ion concentration after the second tumor lung metastasis operation. **(B)** The blood PTH change curve during hospitalization and follow-up in our hospital from May 29, 2016 to January 18, 2024, where the specially marked points are the first blood PTH concentration before parathyroid cancer surgery, the first blood PTH concentration after parathyroid cancer surgery, the first blood PTH concentration before tumor lung metastasis, the first blood PTH concentration after tumor lung metastasis surgery, the second blood PTH concentration before tumor lung metastasis, and the second blood PTH concentration after tumor lung metastasis surgery. The red arrows indicate the time periods of the three surgeries. (Ca2+: normal reference range: 8.5-10.2 mg/dL; 2.12-2.55 mmol/L) (PTH: normal reference range: 15-65 pg/mL; 1.6-6.9 pmol/L).

Before each surgery, the patient’s serum calcium and parathyroid hormone levels remained abnormally elevated, returning to normal after surgery. Routine imaging studies have not revealed further metastatic lesions. At the latest follow-up in 2024, the patient is asymptomatic, with serum calcium and PTH within normal range. She will continue to receive regular biochemical and radiological monitoring, which emphasizes the importance of long-term monitoring in the treatment of parathyroid cancer (**Figure 4**).

## 6 Discussion

PC is a rare but aggressive malignancy, accounting for less than 1% of PHPT cases. Its management is challenging due to overlapping histopathological features with benign adenomas. Typical findings like capsular invasion, vascular invasion, and mitotic activity are not always present, making diagnosis difficult. Intraoperative frozen sections often fail to distinguish PC from benign lesions, underscoring the need for experienced pathologists and multidisciplinary management (8, 9). This particular case underscores both the aggressive nature of PC and the challenges associated with its initial diagnosis, which was only confirmed in this patient following the occurrence of the first pulmonary metastasis.

Certain clinical characteristics may suggest the presence of PC, notwithstanding the associated diagnostic difficulties. Principal indicators encompass persistently elevated PTH levels, severe hypercalcemia, and palpable neck masses (10–12). Notably, PTH levels often exceed five times the upper limit of normal. The combination of these clinical signs with intraoperative observations, such as tumor adherence to adjacent structures, assists surgeons in formulating a presumptive diagnosis of PC and in devising an appropriate surgical management plan. Our preliminary suspicion was informed by the surgical findings, intraoperative frozen section analysis, and markedly elevated preoperative PTH and calcium levels in this patient. Subsequent follow-up evaluations corroborated our initial assessment.

Surgical resection is the primary treatment for PC, addressing both primary and metastatic disease. In this particular patient, leveraging the surgeon’s extensive expertise, we executed an extended resection promptly, thereby reducing the risk of primary tumor recurrence. Nonetheless, the potential for distant metastasis of PC persists. In this context, multiple surgical interventions, including radical resection of the primary tumor and resection of metastatic lesions, were crucial for effective disease control and the prevention of life-threatening hypercalcemia. Considering the limited effectiveness of nonsurgical interventions such as chemotherapy and radiotherapy in treating PC, complete tumor resection remains essential (13, 14). Although pulmonary metastases are rare, their presence, as demonstrated in this case, underscores the importance of long-term surveillance. Routine biochemical monitoring of serum calcium and PTH levels, in conjunction with imaging, is vital for the early detection of recurrence (15, 16). Immunohistochemistry, particularly PTH staining, is crucial in confirming the diagnosis of metastatic PC, particularly in distinguishing it from primary lung malignancies (17).

Molecular markers are of great value in the diagnosis of parathyroid carcinoma. Among them, the mutation of the CDC73 gene and the absence of its protein product parafibromin are the most specific molecular events, which can significantly improve the ability to differentiate from benign parathyroid lesions. Genetic testing and immunohistochemistry not only provide confirmatory evidence when traditional histological diagnosis is uncertain, but can also be used for early screening of family members at high risk. Some markers (such as Cyclin D1, Ki-67, Galectin-3, and specific microRNA expression profiles) are helpful in evaluating the invasiveness and recurrence risk of the tumor. In addition, these molecular information provide a basis for exploring targeted therapy and formulating individualized management strategies, enabling the diagnosis and treatment of parathyroid carcinoma to shift from relying solely on morphology to molecular precision medicine (18–20). Therefore, in this case, we retrieved the parathyroid lesion specimen of the patient from 2016 and added immunohistochemical staining of parafibromin protein. The result showed that parafibromin protein was negative and not expressed in this parathyroid lesion. We used normal parathyroid tissue and breast cancer tissue as positive controls. This further indicates that at that time, the patient should have been diagnosed with parathyroid carcinoma rather than atypical parathyroid adenoma.

Parathyroid carcinoma frequently recurs within two to five years following the initial surgical intervention. The rates of local recurrence range from 33% to 82% at the five-year mark, likely attributable to incomplete resection (21–24). This case aligns with existing literature, as the patient experienced two recurrences within this timeframe. Rigorous postoperative monitoring is necessary, including serum calcium and PTH surveillance, as biochemical relapse often precedes clinical symptoms. Comprehensive screening, including PET/CT, bone scans, and liver imaging, is essential for detecting distant metastases. Utilizing a combination of at least two diagnostic techniques increases the sensitivity for detecting recurrent disease, as demonstrated in a series of 14 reintervention cases, where the Tc-99m sestamibi scan, CT scan, and ultrasonography exhibited sensitivities of 86%, 79%, and 100%, respectively (9).

Long-term surveillance and management of PC require a multidisciplinary approach. Endocrinologists, surgeons, oncologists, and radiologists must work together to provide optimal patient care. Repeated surgeries for resectable metastases, particularly in the lungs and bones, may offer symptomatic relief and improved survival, Lifelong surveillance, with regular imaging and biochemical testing, is essential to promptly detect disease progression and manage recurrences effectively (25). A multimodal approach, involving surgical resection of recurrent and metastatic lesions, remains the cornerstone of treatment, with long-term follow-up required to monitor for further recurrences.

## Data Availability

The raw data supporting the conclusions of this article will be made available by the authors, without undue reservation.
