# Supplementary material for: Case Report: Delayed diagnosis of parathyroid carcinoma and two pulmonary recurrences and metastases
Source: Front Oncol. 2025 Sep 24;15:1581911. doi: 10.3389/fonc.2025.1581911 (PMC12505322; doi:10.3389/fonc.2025.1581911)
Supplement: Supplementary file 1 [file DataSheet1.docx]

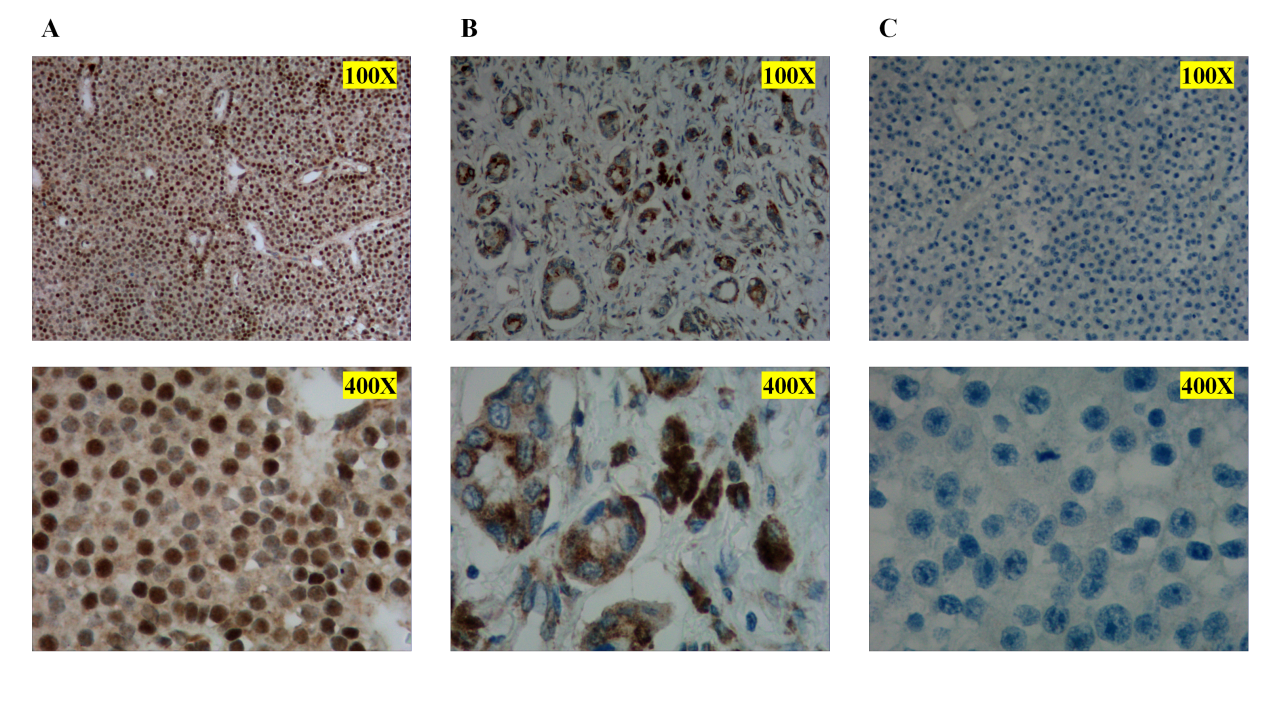


1. Image of normal parathyroid tissue after parafibromin staining. (Top is at 100X magnification, bottom is at 400X magnification) **B.** Image of breast cancer tissue after parafibromin staining. (Top is at 100X magnification, bottom is at 400X magnification) **C.** Image of the patient's parathyroid lesion tissue in 2016 after parafibromin staining. (On the top is a 100X microscope, and below is a 400X microscope)
